# Association of Ultrasonographic Parameters with Subclinical White-Matter Hyperintensities in Hypertensive Patients

**DOI:** 10.1155/2012/616572

**Published:** 2012-09-26

**Authors:** Ioannis Heliopoulos, Dimitrios Artemis, Konstantinos Vadikolias, Grigorios Tripsianis, Charitomeni Piperidou, Georgios Tsivgoulis

**Affiliations:** ^1^Department of Neurology, School of Medicine, Democritus University of Thrace, Alexandroupolis, Greece; ^2^Department of Biostatistics, School of Medicine, Democritus University of Thrace, Alexandroupolis, Greece

## Abstract

*Background and Purpose*. Cerebral white matter hyperintensities (WMHs) are regarded as typical MRI expressions of small-vessel disease (SVD) and are common in hypertensive patients. Hypertension induces pathologic changes in macrocirculation and in microcirculation. Changes in microcirculation may lead to SVD of brain and consequently to hypertensive end-organ damage. This damage is regarded the result of interactions between the macrovascular and microvascular levels. We sought to investigate the association of cerebral WMHs with ultrasonographic parameters of cerebral macrocirculation evaluated by carotid duplex ultrasound (CDU) and transcranial doppler (TCD). *Subjects and Methods*. The study was prospective, cross-sectional and consecutive and included hypertensive patients with brain MRI with WMHs. Patients underwent CDU and TCD. The clinical variables recorded were demographic characteristics (age, gender, race) and vascular risk factors (hypertension, diabetic mellitus, hypercholesterolemia, current smoking, and body mass index). Excluded from the study were patients with history of clinical stroke (including lacunar stroke and hemorrhagic) or transient ischemic attack (either hemispheric or ocular), hemodynamically significant (>50%) extra- or intracranial stenosis, potential sources of cardioembolism, and absent transtemporal windows. WMHs were quantified with the use of a semiquantitative visual rating method. Ultrasound parameters investigated were (1) common carotid artery (CCA) diameter and intima-media thickness, (2) blood flow velocity in the CCA and internal carotid artery (ICA), and (3) blood flow velocity and pulsatility index of middle cerebral artery (MCA). *Results*. A total of 52 patients fulfilled the study inclusion criteria (mean age 71.4 ± 4.5 years, 54% men, median WMH-score: 20). The only two ultrasound parameters that were independently associated with WMH score in multivariate linear regression models adjusting for demographic characteristics and vascular risk factors were increased mean common carotid artery (CCA) diameter (beta = 0.784, SE = 0.272, *P* = 0.006, *R* ^2^ = 23.9%) and increased middle cerebral artery pulsatility index (MCA-PI; beta = 0.262, SE = 0.110, *P* = 0.025, *R* ^2^ = 9.0%). Among all ultrasound parameters the highest AUC (areas under the receiver operating characteristic curve) were documented for MCA-PI (AUC = 0.82, 95% CI = 0.68−0.95, *P* < 0.001) and mean CCA diameter (AUC = 0.80, 95% CI = 0.67−0.92, *P* < 0.001). *Conclusions*. Our study showed that in hypertensive individuals with brain SVD the extent of structural changes in cerebral microcirculation as reflected by WMHs burden is associated with the following ultrasound parameters of cerebral macrocirculation: CCA diameter and MCA-PI.

## 1. Introduction

There is accruing data indicating that white matter hyperintensities (WMHs), the most common imaging biomarker of small-vessel disease (SVD), are longitudinally associated with increased risk of cognitive decline and gait disturbance [1]. Older age and hypertension are the two clinical factors that have been consistently associated with increased burden of WMHs in asymptomatic individuals [2]. The suggested underlying mechanism linking elevated blood pressure levels with SVD includes hypertension-induced structural vessel changes in microcirculation such as lipohyalinosis and fibrinoid degeneration [3]. Hypertension also affects macrocirculation causing arterial remodeling (reflected by increased large-artery diameter) and atheromatosis (reflected by common carotid artery intima-media thickening) both in extra- and proximal intracranial arteries of cerebral circulation [4].

High-resolution B-mode ultrasonography can non-invasively and reliably detect the hypertension-induced structural changes in extracranial macrocirculation that affect common carotid artery intima-media thickness (CCA-IMT), common carotid artery (CCA) diameter, and presence of carotid artery plaques [4, 5]. Transcranial doppler (TCD) also provides additional information about intracranial cerebral hemodynamics and intracranial macrocirculation structural changes (e.g., increased arterial stiffness) using indirect measurements such as pulsatility index (PI) and mean flow velocity (MFV) levels [6].

Limited studies have assessed the relation between WMHs on brain MRI and ultrasound assessments of structural macrocirculation-vessel changes with partly conflicting findings [7–10]. These inconsistent observations may be attributed to different inclusion clinical (e.g., history of previous stroke) and neuroimaging (e.g., presence of concomitant lacunar infarction) criteria, quantification of WMHs by different rating scales, and ultrasound evaluation of different parts of macrocirculation (extracranial versus intracranial) using diverse neurosonology protocols. In view of the former considerations we sought to cross-sectionally investigate the association of ultrasonographic parameters of macrocirculation (evaluated by carotid duplex ultrasound and TCD) with the extent of WMHs on brain MRI in hypertensive individuals without prior history of stroke and no neuroimaging evidence of asymptomatic small- or large-vessel infarction.

## 2. Methods

### 2.1. Study Population

The study was prospective, cross-sectional and consecutive and included all hypertensive patients who have had a brain MRI with white matter disease (white matter changes on MRI in concordance with SVD), referred to the cerebrovascular ultrasound laboratory of our department for assessment of extracranial and intracranial arteries between the years 2006 and 2007.

We excluded patients with: (1) history of clinical stroke (including lacunar stroke and hemorrhagic) or transient ischemic attack either hemispheric or ocular, (2) a >50% stenotic disease of extracranial carotid and/or absence of flow or reversed flow of vertebral arteries on ultrasonography, (3) inadequate temporal windows on TCD permitting acquisition of flow velocities or an asymmetrical middle cerebral artery (MCA) blood flow velocities and/or focal >50% stenosis, (4) cardiogenic sources of emboli, and (5) WMH of non-atherosclerotic etiology.

Clinical variables were recorded on all individuals including demographic characteristics (age, gender, race) and vascular risk factors (hypertension, diabetic mellitus, hypercholesterolemia, current smoking, and body mass index) as previously described [11, 12]. 

### 2.2. Brain MRI and WMH Quantification

Brain MRI was performed in all patients using axial T1-weighted, T2-weighted, fluid-attenuated inversion recovery (FLAIR), and proton-density-weighted scans on 1.0-Tesla MRI scanner (Signal General Electric; GEMS) in the axial and sagittal planes with the slice thickness of 5 mm. We used the previously validated method by Leys et al. to quantify the extent of WMH [13]. This method uses a semiquantitative visual rating of signal hyperintensities on T2-weighted axial images as described in detail in Table 1. All MRI scans were evaluated by the same investigator (DA) who was blinded to the ultrasound parameters and clinical variables of the study population.

### 2.3. Neurosonology Protocol

**Figure 1:**
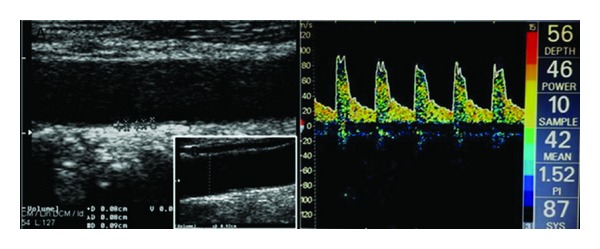
Ultrasound measurements. (A) CCA-IMT. (B) CCA diameter. (C) MCA-PI.

Two experienced in neurosonology investigators (IH, KV) [12, 14] performed the ultrasound investigations within next month after MRI and they were blinded to clinical and MRI data. Carotid ultrasound scanning was performed using an ATL HDI 1500 scanner (Advanced Technologies Laboratories, Bothell, WA, USA) equipped with a 4–7 MHz linear array probe for gray-scale (B-mode), pulsed-wave doppler (PWD), and color doppler flow imaging (CDFI). Our duplex ultrasound examination protocol was previously described [15]. Mean CCA diameter (Figure 1) was computed as follows: mean diameter: (peak-systolic diameter + end-diastolic diameter)/2. 

The degree of stenosis was calculated according to the Consensus Criteria of Society of Radiologists in Ultrasound [16] as previously described [15].

The CCA-IMT was assessed as previously described [15]. The CCA-IMT value was defined as the mean of the right and left IMT of the CCA, calculated from 5 measurements on each side, taken 10 mm proximal to the carotid bifurcation. The lumen/intima leading edge (I line) to media/adventitia leading edge (M line) method, which has been previously validated anatomically [17], was used (Figure 1). Patients with evidence of plaques (defined as a focal structure encroaching into the arterial lumen of at least 0.5mm or 50% of the surrounding IMT value) at the site of CCA-IMT measurements were excluded from further evaluation. The reproducibility of CCA-IMT measurements between and within sonographers in our neurosonology laboratory has been previously established [15]. 

Proximal anterior circulation intracranial arteries were evaluated using a single-channel, 2 MHz TCD (EME/Nicolet Biomedical, Inc, Madison, WI, USA). An insonation depth of 50–60 mm and 60–65 mm was used for the identification of proximal (M1) middle cerebral artery (MCA) and for measurement of flow velocity (FV) and pulsatility index (PI) (Figure 1) and terminal internal carotid artery (TICA) during transtemporal insonation as previously described [14]. The following MFV (mean flow velocity) cutoffs on TCD were used for identification and exclusion of patients with ≥50% stenosis according to SONIA (Stroke Outcomes and Neuroimaging of Intracranial Atherosclerosis) Trial [18] criteria: MCA MFV >100 cm/s, TICA MFV >90 cm/s.

### 2.4. Statistical Analyses

Continuous variables with normal and skewed distribution are presented as mean (SD) or as median (interquartile range (IQR)). Correlation calculations between ultrasound parameters and WMH score were performed by Spearman's correlation coefficient (*r*). The null hypothesis was tested at a level of *P* < 0.05. Univariate and multivariate linear regression models were used to evaluate which ultrasound parameters were independently associated with WMH score (entered as a linear variable in the models) after adjusting for clinical confounders. All factors that contributed to the outcome in the initial univariate analyses at *P* < 0.1 were included in the multivariate linear regression model as candidate variables and then removed by backward stepwise selection procedure. In the final multivariate analyses, statistical significance was achieved if *P* < 0.05. To confirm the robustness of multivariate models, we repeated all multivariate analyses using a forward procedure. For the evaluation of the predictive value of ultrasound parameters to identify patients with high burden of WMH score, the area under the receiver operating characteristic (ROC) curve (AUC) was calculated and corresponding 95% CI was computed. Accuracy parameters including sensitivity, specificity, positive predictive value, negative predictive value and overall accuracy were also calculated. Multivariate logistic regression models were used to identify independent associations between ultrasound parameters and high WMH score burden (entered as a binary variable in the models). Statistical analyses were performed using the SPSS Version 19, Chicago, IL, USA.

## 3. Results

**Figure 2:**
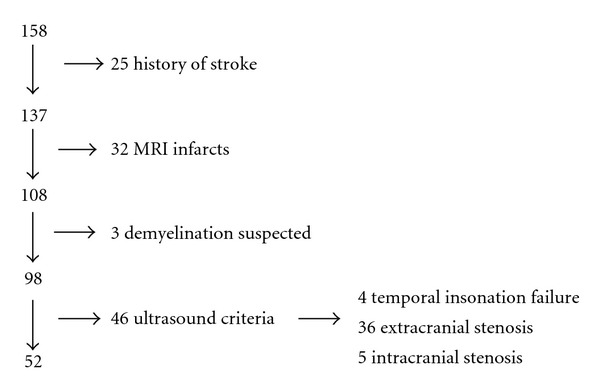
Selection procedure of included patients.

 Of the 158 patients considered, 52 (mean age 71.4 ± 4.5 years, 54% men) met the inclusion criteria (Figure 2). Demographic characteristics and vascular risk factors are summarized in Table 2. All measured ultrasound parameters of extra- and intracranial macrocirculation are displayed in Table 3. CCA-IMT mean and CCA-Dmean (common carotid artery mean diameter) were 0.84 mm and 0.65 cm, respectively. Males had greater CCA-Dmean (0.68 ± 0.01 cm) than females (0.61 ± 0.06; *P* = 0.003).

WMH score ranged from 10 to 41, with a mean score of 22.1 ± 9.1 (median score, 20). The correlation of ultrasound parameters with WMH is shown in Table 4. CCA diameters (CCA-Dsys (common carotid artery diameter in the systolic phase), CCA-Ddia (common carotid artery in the diastolic phase), and CCA-Dmean (mean common carotid artery diameter) significantly correlated with WMH (Spearman's correlation coefficient: *r* = 0.501–0.508, *P* < 0.001 for all three correlations). The former association still retained its statistical significance (*P* < 0.001) even after adjustment for gender.

No association of CCA-IMT mean (*r* = 0.228, *P* = 0.104) and CCA-MFV (*r* = −0.134, *P* = 0.342, resp.) was documented. ICA-EDV and ICA-MFV were negatively correlated to WMH score (*r* = −0.324, *P* = 0.019 and *r* = −0.363, *P* = 0.008 resp.). A positive correlation of WMH score with of MCA-PI (*r* = 0.516, *P* < 0.001) was observed, but not with of MCA-MFV (*r* = −0.041, *P* = 0.819). 

In the initial univariate linear regression analysis, the following three ultrasound parameters were associated with WMH score in descending order: mean CCA diameter (beta = 1.342, SE = 0.302, *P* < 0.001), MCA-PI (beta = 0.317, SE = 0.132, *P* = 0.022), and extracranial ICA MFV (beta = −0.008, SE = 0.004, *P* = 0.038). Finally, in the multivariate linear regression models (adjusting for demographic characteristics and vascular risk factors), only two ultrasound parameter were independently associated with WMH score: increased mean CCA diameter (beta = 0.784, SE = 0.272, *P* = 0.006, *R* ^2^ change = 23.9%) and increased MCA-PI (beta = 0.262, SE = 0.110, *P* = 0.025, *R* ^2^ change = 9.0%).

The median value of WMH score (20 points) was selected as the cut-off point to subdivide patients into two groups with high (>20 points) and low (≤20 points) WMH score. No significant differences in any of the patients' baseline characteristics were found between the two groups of patients (data not shown). Patients with high WMH score (*n* = 26) had higher CCA-Dmean (*P* = 0.001) and MCA-PI (*P* = 0.003) when compared to the low WMH score groups. Conversely, CCA-PSV (*P* = 0.024), ICA-EDV (*P* = 0.009) and ICA-MFV (*P* = 0.005) were lower in the high WMH score group.

For the evaluation of the diagnostic significance of these four ultrasound parameters for prediction of high WMH score (>20), the area under ROC curve (AUC) was computed (Table 5). The highest AUCs were documented for MCA-PI (AUC = 0.82, 95% CI = 0.68–0.95, *P* < 0.001) and CCA-Dmean (AUC = 0.80, 95% CI = 0.67–0.92, *P* < 0.001). Clinically important cut-off points for all these parameter *s* were also determined by the ROC curve analysis (Table 5). The corresponding cutoffs with the highest overall accuracy for CCA-Dmean (77%) and MCA-PI (83%) were 0.666 cm and 1.063, respectively.

In multivariate logistic regression analysis, after adjustment for demographic characteristics including gender and vascular risk factors, CCA-Dmean and MCA-PI remained the significant independent predictors for high WMH-score; high CCA-Dmean values (≥0.666 cm) yielded an odds ratio of WMH score >20 points of 15.94 (95% CI = 3.23–78.59, *P* < 0.001). Similarly high MCA-PI values (≥1.063) yielded an odds ratio of WMH score >20 points of 6.90 (95% CI = 1.80–26.39, *P* = 0.005).

## 4. Discussion

Our cross-sectional study showed that two ultrasound parameters of macrocirculation are associated with the extent of structural changes in microcirculation as reflected by WMH burden in a sample of hypertensive individuals without prior history of stroke and no neuroimaging evidence of asymptomatic cerebral infarction. More specifically, higher MCA-PI and larger mean CCA diameter were independently associated with increased WMH-score after adjustment for demographic and clinical characteristics. The former two ultrasound parameters were also able to reliably discriminate individuals with high WMH score. In contrast, we failed to document any relationship between CCA-IMT (an established marker of generalized atherosclerosis) [5] and the number of white-matter lesions.

We observed that increased CCA diameter was linearly associated with greater WMH score on brain MRI. To the best of our knowledge, there is no prior study evaluating the relationship of white-matter lesions as well as symptomatic or clinically silent brain infarctions with CCA diameter. The available literature focuses on CCA remodeling and indicates that thickening of the arterial wall due to hypertension may be accommodated by outward remodeling of the vessel, thereby preserving the luminal area [19]. Interestingly, the Cardiovascular Health Study has shown that increased hypertension load quantified by left ventricular mass was strongly associated with CCA compensatory remodeling leading to larger CCA diameter [20]. Diffuse diameter enlargement may also occur as a physiologic response to increased blood flow or as a change in ageing [21]. However it should be noted that the documented association of increased CCA diameter with higher WMH score was independent of age and other demographic or clinical variables in our study population. Consequently, it may be postulated that the observed association of larger CCA diameter with higher load of small-vessel disease indicates that the remodeling of macrocirculation (increase in CCA diameter) correlates to microcirculation remodeling (arteriolar rarefaction).

We also recorded an independent association between increased MCA-PI and more extensive WMHs on brain MRI. This observation is in line with a previous North-American study that assessed MRI manifestations of small-vessel disease with TCD indices and also found that MCA-PI positively correlated to MRI scores for hemispheric small-vessel disease [9]. However, it should be acknowledged that the two reports studied different populations. More specifically, the former study included patients with acute cerebral ischemia (transient ischemic attack or ischemic stroke) and hypertension was prevalent in almost half of the study population. PI is an index of a combined change in vascular resistance and compliance of large cerebral arteries [22], with the vessels residing outside the brain having the greatest impact on parenchymal blood flow. More specifically, extraparenchymal arteries and arterioles account for 2/3 of the vascular resistance, while intracerebral arterioles and capillaries account for the remaining 1/3 [23]. Therefore the increasing downstream resistance, which is reflected by elevated PI, is mainly the result of the changes in mechanoelastic properties of large intracranial vessel and reduced compliance of these arteries [24]. Recent data indicate that measures of large-artery stiffness are closely related to the effects of microvascular changes in the brain (including leukoaraiosis) and kidney of hypertensive individuals [25]. The former considerations lend support to our assumption that hypertension-induced structural changes in intracranial macrocirculation (reflected by increased MCA-PI) are associated with greater burden of cerebral microvascular disease (reflected by higher WMH score).

Previous reports have yielded conflicting results regarding the potential association between CCA-IMT and WMHs on brain MRI. A French [7] and a Japanese [10] study showed that CCA-IMT was related to the extent of WHMs on brain MRI of hypertensive individuals. Conversely, a Scottish group of investigators failed to reproduce the former association in healthy volunteers without prior history of stroke [8]. We also documented no correlation between CCA-IMT and WMHs. Notably, Japanese investigators after carrying out a multifractal analysis to examine the microstructural changes of the deep white-matter in apparently normal T2-weighted MR images in elderly subjects (without any evidence of atherosclerotic risk factors) also did not detect any association of CCA-IMT with white-matter structure [26]. Consequently, it may be argued that CCA-IMT may be a marker of large-vessel rather than small-vessel disease damage. Recent studies [27, 28], documenting significantly greater carotid artery intima-media thickening in patients with ischemic stroke due to large-artery atherosclerosis in comparison to patients with lacunar infarction, concur the former hypothesis. Nevertheless, it should be acknowledged that the discrepant results across different studies may be related to different sample sizes (ranging from 49 [10] to 198 [7] individuals), differences in demographic characteristics including mean age (ranging from 59 [10] to 78 [8] years) and race (Caucasians [7, 8] versus Asians [10]) and different methodology for CCA-IMT estimation (manual [8, 10] versus semiautomatic [7] measurements). Finally, a type II error cannot be ruled out given the moderate sample size of our study population.

Our study has limitations including the cross-sectional design that does not allow us to ascertain any causal relationship between ultrasound parameters and the extent of WMH burden. Furthermore the presence and extent of microbleeds on brain MRI, which have also been established as an imaging biomarker of small-vessel disease [1], were not evaluated. Finally, our sample size was modest, and therefore potential residual confounding in the observed associations both in the multivariate linear and logistic regression models cannot be reliably excluded. A further study with large sample size is needed to strengthen our results. On the other hand, it should be noted that we used strict inclusion and exclusion criteria to investigate a population of essentially hypertensive individuals without prior history of cerebrovascular disease, no imaging evidence of asymptomatic infarction, no ultrasound evidence of hemodynamically significant stenosis, and after performing a diagnostic workup that excluded potential sources of cardioembolism. Also, to the best of our knowledge this is the first study combining ultrasound parameters of both extra- and intracranial macrocirculation.

In conclusion, our pilot study points to an independent association of higher MCA-PI and larger mean CCA diameter with more extensive WMH burden on brain MRI in hypertensive, asymptomatic patients. Further observational studies are needed to verify this potentially intriguing association in a longitudinal fashion and to investigate whether these ultrasound parameters may constitute imaging biomarkers that could predict extensive small-vessel disease in cerebral circulation leading to cognitive impairment and gait disturbance.

## Figures and Tables

**Table 1:** Semiquantitative visual rating of white matter hyperintensities.

Lesions	Score
Periventricular hyperintensities (0–6)	
Frontal caps (0–2)	0: absent
Occipital caps (0–2)	1: ≤5 mm
Lateral ventricle bands (0–2)	2: ≥5 mm

White matter hyperintensities (0–24)	
Frontal (0–6)	0: no abnormality
Parietal (0–6)	1: <3 mm; *n* ≤ 5
Occipital (0–6)	2: <3 mm; *n* > 6
Temporal (0–6)	3: 4–10 mm; *n* ≤ 5
	4: 4 mm; *n* > 6
	5: >11 mm; *n* > 1
	6: confluent

Basal ganglia hyperintensities (0–30)	
Caudate nucleus (0–6)	0: no abnormality
Putamen (0–6)	1: <3 mm; *n* ≤ 5
Globus pallidus (0–6)	2: <3 mm; *n* > 6
Thalamus (0–6)	3: 4–10 mm; *n* ≤ 5
Internal capsule (0–6)	4: 4 mm; *n* > 6
	5: >11 mm; *n* > 1
	6: confluent

Infratentorial foci of hyperintensities (0–24)	
Cerebellum (0–6)	0: no abnormality
Mesencephalon (0–6)	1: <3 mm; *n* ≤ 5
Pons (0–6)	2: <3 mm; *n* > 6
Medulla (0–6)	3: 4–10 mm; *n* ≤ 5
	4: 4 mm; *n* > 6
	5: >11 mm; *n* > 1
	6: confluent

**Table 2:** Baseline characteristics of the study population (*n* = 52).

Characteristic	
Mean age, years (SD)	71.4 (4.5)
Male sex, % (*n*)	54% (26)
Mean body mass index, Kg/m^2^ (SD)	27.5 (2.7)
Hypertension, % (*n*)	100% (52)
Diabetes Mellitus, % (*n*)	21% (*n* = 11)
Hypercholesterolemia, % (*n*)	25% (*n* = 13)
Current smoking, % (*n*)	31% (*n* = 16)

**Table 3:** Ultrasound assessment of extra- and intracranial macrocirculation.

Variable	Right	Left
Extracranial circulation		
CCA-Dsys, cm (mean, SD)	0.68 (0.09)	0.65 (0.10)
CCA-Ddia, cm (mean, SD)	0.65 (0.09)	0.62 (0.09)
CCA-Dmean, cm (mean, SD)	0.66 (0.09)	0.63 (0.10)
CCA-PSV, cm/sec (mean, SD)	38.9 (9.6)	40.7 (8.7)
CCA-EDV, cm/sec (mean, SD)	7.3 (3.8)	9.0 (3.9)
CCA-MFV, cm/sec (mean, SD)	17.8 (5.1)	19.6 (5.2)
ICA-PSV, cm/sec (mean, SD)	53.7 (18.0)	55.2 (12.5)
ICA-EDV, cm/sec (mean, SD)	16.0 (8.6)	15.0 (6.6)
ICA-MFV, cm/sec (mean, SD)	28.6 (9.9)	28.3 (7.8)
CCA-IMTmean, mm (mean, SD)	0.8 (0.2)	0.9 (0.2)

Intracranial circulation		
MCA-MFV, cm/sec (mean, SD)	35.4 (10.5)	35.6 (12.0)
MCA PI (mean, SD)	1.15 (0.25)	1.13 (0.24)

CCA-Dsys: (CCA diameter, peak-systolic), CCA-Ddia: (CCA diameter, end-diastolic), CCA-Dmean: (CCA diameter mean), CCA-PSV: (CCA peak-systolic flow velocity, CCA-EDV: (CCA end-diastolic flow velocity, CCA-MFV: (CCA mean-flow velocity), ICA-PSV: (ICA peak-systolic flow velocity), ICA-EDV: (ICA end-diastolic flow velocity), ICA-MFV: (ICA mean-flow velocity), MCA-MFV: (MCA mean-flow velocity), MCA PI: (MCA pulsatility index).

**Table 4:** Correlation of ultrasound parameters with white-matter hyperintensities on brain MRI.

Variable	Spearman's *r*	*P*
Extracranial circulation		
CCA-Dsys	+0.501	<0.001
CCA-Ddia	+0.506	<0.001
CCA-Dmea	+0.508	<0.001
CCA-PSV	−0.256	0.067
CCA-EDV	−0.205	0.144
CCA-MFV	−0.134	0.342
ICA-PSV	−0.135	0.341
ICA-EDV	−0.324	0.019
ICA-MFV	−0.363	0.008
CCA-IMT	+0.228	0.104

Intracranial circulation		
MCA-MFV	−0.041	0.819
MCA-PI	+0.516	<0.001

**Table 5:** Accuracy parameters of different cutoffs of ultrasound parameters for prediction of high white matter hyperintensities score (>20).

Ultrasound parameter	Cutoff	Sens.	Spec.	PPV	NPV	Overall accuracy	Cohen's *κ*	AUC (95% CI)	*P*
*CCA-Dmean* (cm)								**0.80** **(0.67–0.92)**	**<0.001**
	≥0.576	96%	46%	64%	92%	71%	0.423		
	*≥0.666*	*65%*	*89%*	*85%*	*72%*	*77%*	*0.538*		
	≥0.705	39%	96%	91%	61%	67%	0.346		

CCA-PSV (cm/sec)								0.69 (0.54–0.83)	0.022
	≤34.663	39%	92%	83%	60%	65%	0.308		
	≤38.470	62%	69%	67%	65%	65%	0.308		
	≤45.315	85%	39%	58%	74%	62%	0.231		

ICA-MFV (cm/sec)								0.74 (0.60–0.88)	0.003
	≤19.415	27%	100%	100%	58%	64%	0.269		
	≤27.545	77%	77%	77%	77%	77%	0.538		
	≤35.990	92%	31%	57%	80%	62%	0.231		

*MCA-PI*								**0.82** **(0.68–0.95)**	**<0.001**
	*≥1.063*	*100%*	*68%*	*74%*	*100%*	*83%*	*0.671*		
	≥1.183	60%	86%	80%	70%	74%	0.469		
	≥1.445	25%	91%	71%	57%	60%	0.164		

Sens.: sensitivity, spec.: specificity, PPV: positive predictive value, NPV: negative predictive value, AUC: area under the receiver operating characteristic curve, CI: confidence interval.
